# Numerical Study of Natural Convection within a Wavy Enclosure Using Meshfree Approach: Effect of Corner Heating

**DOI:** 10.1155/2014/842401

**Published:** 2014-01-28

**Authors:** Sonam Singh, R. Bhargava

**Affiliations:** Department of Mathematics, Indian Institute of Technology Roorkee, Uttarakhand 247667, India

## Abstract

This paper presents a numerical study of natural convection within a wavy enclosure heated via corner heating. The considered enclosure is a square enclosure with left wavy side wall. The vertical wavy wall of the enclosure and both of the corner heaters are maintained at constant temperature, *T*_*c*_ and *T*_*h*_, respectively, with *T*_*h*_ > *T*_*c*_ while the remaining horizontal, bottom, top and side walls are insulated. A penalty element-free Galerkin approach with reduced gauss integration scheme for penalty terms is used to solve momentum and energy equations over the complex domain with wide range of parameters, namely, Rayleigh number (Ra), Prandtl number (Pr), and range of heaters in the *x*- and *y*-direction. Numerical results are represented in terms of isotherms, streamlines, and Nusselt number. It is observed that the rate of heat transfer depends to a great extent on the Rayleigh number, Prandtl number, length of the corner heaters and the shape of the heat transfer surface. The consistent performance of the adopted numerical procedure is verified by comparison of the results obtained through the present meshless technique with those existing in the literature.

## 1. Introduction

Natural convection within closed cavities with different fluids has attracted considerable attention of many researchers [1–6] due to its immense applications in engineering such as the cooling of electronic devices, refrigerators, room ventilating, heat exchangers, and solar collectors. A good amount of literature is available for convection patterns in differentially heated rectangular enclosures. The basic benchmark study related to this subject was reported by De Vahl Davis [2] and De Vahl Davis and Jones [3]. Later, many authors [4–6] studied the impact of different boundary conditions on convection phenomenon within an enclosure. In these studies, different shapes of heat transfer surfaces were not considered.

However, the analysis of natural convection heat transfer with different shapes of heat transfer surfaces (e.g., wavy surfaces or rough surfaces) is extremely important in many applications where natural convection is the only feasible mode of cooling. The shape of the surface influences the performances of the heat transfer equipment. Hence, there has been considerable interest in the intentional roughening of the surface to enhance the heat transfer. This small scale roughness may be represented by periodic functions (sine and cosines). A literature review of free convection heat transfer from vertical surfaces with surface roughness elements in continuum fluid is provided by Bhavnani and Bergles [7]. Varol and Oztop [8] performed a comparative numerical study on natural convection in wavy and flat plate solar collectors and it was observed that heat transfer rate is higher for wavy collector as compared to the flat plate collector. Dalal and Das [9] discussed laminar natural convection in an inclined complicated wavy cavity with spatially variable wall temperature. Saha [10] analyzed magnetoconvection inside a sinusoidal corrugated enclosure where bottom surface is heated with discrete isoflux using finite element method.

Besides, the linear distribution of temperature along the heated surface behavior of many engineering systems such as building construction elements with passive solar heating, solar pond, and liquid fuel storage tank may be characterized with concentrated heat source on one side or having partial heaters. Chu et al. [11] investigated the natural convection in an enclosure with a partial heater located at the left vertical wall and the cooling condition is applied on right vertical wall, both experimentally and numerically. Zhao et al. [12] analyzed double-diffusive natural convection in a porous enclosure which is partially heated from one side. It was observed that both the location and length of the heater have considerable impact on temperature and flow field. More work on this can be found in the literature [13–17].

In the present study, our aim is to investigate the effect of corner heaters on flow field and temperature distribution within an enclosure which has complex geometry due to wavy side wall. An advanced meshfree numerical technique, element free Galerkin method (EFGM), is used to solve the nonlinear mathematical model of the problem. Although finite element method and finite difference methods are quite efficient and general techniques, they behave poorly for the simulation of problems where complex geometry is involved, due to tedious and expensive meshing, remeshing procedure. To overcome these difficulties, a number of meshfree methods such as element free Galerkin method, meshless local Petrov-Galerkin method, and smooth particle hydrodynamics method have been proposed.

Nikfar and Mahmoodi [18] applied meshless local Petrov-Galerkin method for the analysis of natural convection of nanofluids inside a cavity having wavy side walls. The element free Galerkin method has also been successfully used to solve various problems in different areas such as heat transfer [19] and fracture mechanics [20]. Recently, Singh and Bhargava [21] have applied EFGM for the simulation of an unsteady micropolar squeeze film flow.

Hence, in the present study EFGM has been used as a tool to solve the coupled, nonlinear partial differential equations governing the flow inside a wavy enclosure. Obtained results are compared with benchmark results and excellent agreement has been observed between them.

## 2. Mathematical Model and Definition of the Problem

**Figure 1:**
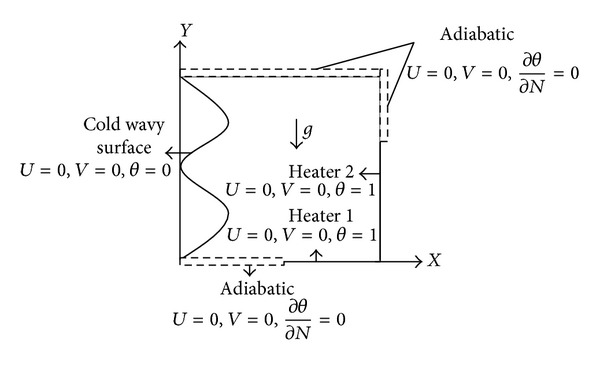
Geometrical model of the problem.

A schematic view of the two-dimensional enclosure with one wavy side wall is depicted in Figure 1. The left wavy wall of the enclosure is described by

(1) $$\begin{array}{c} x=a\;\sin^{2}\left(\frac{n\pi y}{L}\right). \end{array}$$

The wavy wall of the enclosure is maintained at a constant temperature *T*_*c*_ and its temperature is lower than that of the corner heaters, maintained at constant temperature *T*_*h*_. The remaining top, bottom, and side walls are insulated. All the physical properties of the fluid are assumed to be constant except the density which is determined according to Boussinesq approximation, primarily used in the field of buoyancy-driven flow. It is assumed that viscous dissipation and radiation effects are negligible and gravity acts in the negative *y*-direction. For a two-dimensional flow of an incompressible Newtonian fluid in steady state regime, the governing continuity, momentum, and energy equations can be obtained with the following dimensionless variables [4, 5]:

(2) $$\begin{array}{c} X=\frac{x}{L},\;\;Y=\frac{y}{L},\;\;\left(U,V\right)=\frac{\left(u,v\right)L}{\alpha },\\ \theta =\frac{T-T_{c}}{T_{h}-T_{c}},\;\;A=\frac{a}{L},\\ P=\frac{pL^{2}}{\rho \alpha^{2}},\;\;\text{Pr}=\frac{\upsilon }{\alpha },\;\;\text{Ra}=\frac{g\beta \left(T_{h}-T_{c}\right)L^{3}\text{Pr}}{\upsilon^{2}}. \end{array}$$

The governing equations are written as

(3) $$\begin{array}{c} \frac{\partial U}{\partial X}+\frac{\partial V}{\partial Y}=0, \end{array}$$

(4) $$\begin{array}{c} U\frac{\partial U}{\partial X}+V\frac{\partial U}{\partial Y}=-\frac{\partial P}{\partial X}+\text{Pr}\left(\frac{\partial^{2}U}{\partial X^{2}}+\frac{\partial^{2}U}{\partial Y^{2}}\right), \end{array}$$

(5) $$\begin{array}{c} U\frac{\partial V}{\partial X}+V\frac{\partial V}{\partial Y}=-\frac{\partial P}{\partial Y}+\text{Pr}\left(\frac{\partial^{2}V}{\partial X^{2}}+\frac{\partial^{2}V}{\partial Y^{2}}\right)+\text{Ra}\;\text{Pr}\;\theta , \end{array}$$

(6) $$\begin{array}{c} U\frac{\partial \theta }{\partial X}+V\frac{\partial \theta }{\partial Y}=\frac{\partial^{2}\theta }{\partial X^{2}}+\frac{\partial^{2}\theta }{\partial Y^{2}}. \end{array}$$

The physical boundary conditions as shown in geometric model (Figure 1) can be defined as follows.

On the wavy wall:

(7) $$\begin{array}{c} U=0,\;\;V=0,\;\;\theta =0. \end{array}$$

On the adiabatic walls:

(8) $$\begin{array}{c} U=0,\;\;V=0,\;\;\frac{\partial \theta }{\partial N}=0. \end{array}$$

On the corner heaters:

(9) $$\begin{array}{c} U=0,\;\;V=0,\;\;\theta =1. \end{array}$$

### 2.1. Nusselt Number Evaluation

The heat transfer rate evaluation is the most important for many engineering applications. Heat transfer rates on the surface of isothermal horizontal, vertical heaters, and cold wavy surface are computed by local Nusselt number. Mean Nusselt number is calculated by averaging local nusselt number along the cold wavy side of the enclosure.

The local nusselt number along the cold wavy surface is given as

(10) $$\begin{array}{c} {\text{Nu}}_{\text{CS}}=-{\frac{\partial \theta }{\partial X}|}_{X=A\sin^{2}(n\pi Y)}. \end{array}$$

The heat transfer rate in terms of local Nusselt number along the horizontal (HS1) or vertical (HS2) heaters is expressed as

(11) $$\begin{array}{c} {\text{Nu}}_{\text{HS}1}=-{\frac{\partial \theta }{\partial Y}|}_{Y=0},\\ {\text{Nu}}_{\text{HS}2}=-{\frac{\partial \theta }{\partial X}|}_{X=1}. \end{array}$$

The mean nusselt number is computed along the cold wavy surface and is given as follows,

(12) $$\begin{array}{c} \text{Nu}=\overset{1}{\underset{0}{\int }}{\text{Nu}}_{\text{CS}}dY. \end{array}$$

## 3. Solution Methodology and Postprocessing

The momentum and energy conservation equations are solved using a meshfree numerical technique known as element free Galerkin method. The continuity equation is used as a constraint due to mass conservation and pressure distribution [4, 5]. The penalty approach [24, 25] is employed to impose this constraint. In order to solve momentum equations (4)-(5), pressure is eliminated by a penalty parameter and the incompressibility condition given in (3) results

(13) $$\begin{array}{c} P=-\gamma \left(\frac{\partial U}{\partial X}+\frac{\partial V}{\partial Y}\right). \end{array}$$

For large values of penalty parameter *γ*, the continuity equation (3) is automatically satisfied. Using (13), the momentum conservation equations (4)-(5) result in

(14) $$\begin{array}{c} U\frac{\partial U}{\partial X}+V\frac{\partial U}{\partial Y}=\gamma \frac{\partial }{\partial X}\left(\frac{\partial U}{\partial X}+\frac{\partial V}{\partial Y}\right)+\text{Pr}\left(\frac{\partial^{2}U}{\partial X^{2}}+\frac{\partial^{2}U}{\partial Y^{2}}\right), \end{array}$$

(15) U∂V∂X+V∂V∂Y=γ∂∂Y(∂U∂X+∂V∂Y)+Pr(∂2V∂X2+∂2V∂Y2)+Ra Pr θ.

Now, the system of simultaneous partial differential equations (6), (14), and (15) is solved numerically using element free Galerkin method.

### 3.1. Meshfree Method

Numerical simulation of fluid flow problems within complex geometries is a computational challenge. Although finite element method (FEM) is one of the advanced and general numerical technique, meshing, remeshing of complex geometries is quite difficult and expensive in FEM. To overcome these problems, a meshfree numerical technique element free Galerkin method is used for the numerical simulation. The essence of meshfree techniques is that, instead of a predefined mesh, they use only a set of nodes scattered in the whole problem domain without any fixed connectivity which is quite easy process as compared to the tedious meshing procedure in FEM.

### 3.2. Element-Free Galerkin Method

**Figure 2:**
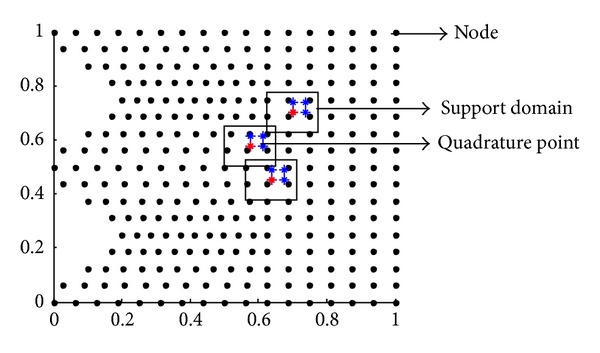
Selection of support domain for a quadrature point.

The element free Galerkin method (EFGM) requires moving least square (MLS) interpolation functions to approximate an unknown function. The MLS approximant requires only a set of nodes for its construction and is made up of these components: a compact support weight function associated with each node, a polynomial basis function, and a set of coefficients that depends on node position. The weight function is nonzero over a small neighborhood of a particular quadrature point or evaluation point, in which small neighborhood area is called support domain of the quadrature point. A view for selecting support domain of a quadrature point is shown in Figure 2.

Using MLS approximation, the unknown field variable *u*(*x*, *y*) is approximated over the two-dimensional domain *Ω* as (details can be seen in [26])

(16) $$\begin{array}{c} u\left(x,y\right)\approx u^{h}\left(x,y\right)=\overset{m}{\underset{j=1}{\sum }}p_{j}\left(x,y\right)a_{j}\left(x,y\right)=p^{T}\left(X\right)a\left(X\right), \end{array}$$

where *X* = (*x*, *y*), *m* is the number of terms in the basis, *p*_*j*_(*x*, *y*) is the monomial basis function, *a*_*j*_(*x*, *y*) is the nonconstant coefficient functions, and *p* ^*T*^(*X*) = [1 *x* *y*].

The coefficients *a*_*j*_(*x*, *y*) are determined by minimizing the functional *J*(*X*) given by

(17) $$\begin{array}{c} J\left(X\right)=\overset{n^{*}}{\underset{I=1}{\sum }}{W\left(X-X_{I}\right)\left\{\overset{m}{\underset{j=1}{\sum }}p_{j}\left(X_{I}\right)a_{j}\left(X\right)-u_{I}\right\}}^{2}, \end{array}$$

where *W*(*X* − *X*_*I*_) is a weight function which is nonzero over a small domain, called support domain, and n* is the number of nodes that are included in the support domain of a point *X*. The minimization of *J*(*X*) with respect to *a*(*X*) leads to the following set of equations:

(18) $$\begin{array}{c} a\left(X\right)=A^{-1}\left(X\right)B\left(X\right)U_{s}, \end{array}$$

where *A* and *B* are given as

(19) A(X)=∑I=1n∗W(X−XI)p(XI)pT(XI),B(X)=[W(X−X1)p(X1),W(X−X2)p(X2),…,W(X−Xn)p(Xn∗)],Us=[u1,u2,…,un∗].

Substituting (19) in (16), the MLS approximant is obtained as

(20) $$\begin{array}{c} u^{h}\left(X\right)=\overset{n^{*}}{\underset{I=1}{\sum }}\Phi_{I}\left(X\right)u_{I}=\Phi \left(X\right)u, \end{array}$$

where the shape function Φ_*I*_(*X*) is defined by

(21) $$\begin{array}{c} \Phi_{I}\left(X\right)=\overset{m}{\underset{j=1}{\sum }}p_{j}\left(X\right){\left(A^{-1}\left(X\right)B\left(X\right)\right)}_{jI}=p^{T}A^{-1}B_{I}. \end{array}$$

#### 3.2.1. Weight Function Description

The choice of weight function affects the resulting approximation *u* ^*h*^(*X*) in EFGM and other meshless methods. In EFGM, the continuity of MLS approximants is governed by the continuity of weight function. Singh et al. [25] have studied these weight functions and reported that cubic spline weight function gives more accurate results as compared to others. Therefore, in the present work, cubicspline weight function has been used.

#### 3.2.2. Cubicspline Weight Function

Consider the following:

(22) $$\begin{array}{c} W\left(r-r_{I}\right)=W\left(r\right)=\left\{\begin{array}{cc} \frac{2}{3}-4r^{2}+4r^{3} & r\leq \frac{1}{2}\\ \frac{4}{3}-4r+4r^{2}-\frac{4}{3}r^{3} & \frac{1}{2}<r\leq 1\\ 0 & r>1 \end{array}\right\}, \end{array}$$

where *r* − *r*_*I*_ = ||*X* − *X*_*I*_||/*d*_*m*_*I*__.

The shape of the support domain can be circular or rectangular or both, but rectangular support domain is more general, and therefore is used in the present simulation. In rectangular support domain, the weight function at any given point can be calculated as

(23) $$\begin{array}{c} W\left(X-X_{I}\right)=W\left(r_{xI}\right)\cdot W\left(r_{ryI}\right). \end{array}$$

where *r*_*I*_ = ||*X* − *X*_*I*_||/*d*_*m*_*I*__, *r*_*x*_*I*__ = |*x* − *x*_*I*_|/*d*_*mx*_*I*__, *r*_*y*_*I*__ = |*y* − *y*_*I*_|/*d*_*my*_*I*__, *d*_*mx*_*I*__ = *d*_max⁡_ *C*_*x*_*I*__ and *d*_*my*_*I*__ = *d*_max⁡_ *C*_*y*_*I*__, *d*_*mx*_*I*__, *d*_*my*_*I*__ are the sizes of support domain in the *x*- and *y*-direction. *d*_max⁡_ is a scaling parameter, and *C*_*x*_*I*__, *C*_*y*_*I*__ are the distances to the nearest neighbors in the *x*- and *y*-direction, respectively. The size of the support domain at a particular node *I* is only controlled by scaling parameter since the distance between nearest neighbors for an evaluation point (or quadrature point) remains unchanged for a given nodal data distribution. The minimum value of *d*_max⁡_ should be greater than 1 so that *n* > *m*, and the maximum value of *d*_max⁡_ should be such that it preserves the local character of MLS approximation. It has been shown by Singh [27] that 1.0 < *d*_max⁡_ < 1.5 is the optimum range of scaling parameter for heat transfer problems. In the present simulation *d*_max⁡_ has been fixed as 1.2.

### 3.3. Variational Formulation

The weighted integral form of (14), (15), and (6) over the entire problem domain can be written as

(24) ∫Ωw1(U∂U∂X+V∂U∂Y−γ∂∂X(∂U∂X+∂V∂Y)−Pr(∂2U∂X2+∂2U∂Y2))=0,∫Ωw2(U∂V∂X+V∂V∂Y−γ∂∂Y(∂U∂X+∂V∂Y)−Pr(∂2V∂X2+∂2V∂Y2)−Ra Pr θ)=0,∫Ωw3(U∂θ∂X+V∂θ∂Y−∂2θ∂X2−∂2θ∂Y2)=0,

where *w*_1_, *w*_2_, *w*_3_ are arbitrary test functions and may be viewed as the variation in *U*, *V*, *θ*, respectively.

### 3.4. Element Free Galerkin Model and Imposition of Boundary Conditions

The element free Galerkin model of (24) is obtained by substituting MLS approximation for the unknown field variables *U*, *V*, *θ* using (20)-(21) and can be obtained as

(25) $$\begin{array}{c} U=\overset{n^{*}}{\underset{I=1}{\sum }}\Phi_{I}f_{I},\;\;V=\overset{n^{*}}{\underset{I=1}{\sum }}\Phi_{I}F_{I},\;\;\theta =\overset{n^{*}}{\underset{I=1}{\sum }}\Phi_{I}\Theta_{I}. \end{array}$$

Since MLS shape functions do not satisfy the kronecker delta property, so we cannot directly impose the essential boundary conditions. To remove this problem, different numerical techniques have been proposed to enforce the essential boundary conditions in EFG method such as Lagrange multiplier method and the penalty method. In the present simulation penalty method is applied.

### 3.5. Penalty Method

Using penalty method to enforce the essential boundary conditions, the variational form is written as

(26) ∫Ωw1(U∂U∂X+V∂U∂Y−γ∂∂X(∂U∂X+∂V∂Y)−Pr⁡(∂2U∂X2+∂2U∂Y2))+∫Γ1α¯w1(U−UΓ1)dΓ+∫Γ2α¯w1(U−UΓ2)dΓ+∫Γ3α¯w1(U−UΓ3)dΓ+∫Γ4α¯w1(U−UΓ4)dΓ=0,∫Ωw2(U∂V∂X+V∂V∂Y−γ∂∂Y(∂U∂X+∂V∂Y)−Pr⁡(∂2V∂X2+∂2V∂Y2)−Ra Pr θ)+∫Γ1α¯w2(V−VΓ1)dΓ+∫Γ2α¯w2(V−VΓ2)dΓ+∫Γ3α¯w2(V−VΓ3)dΓ+∫Γ4α¯w2(V−VΓ4)dΓ=0,∫Ωw3(U∂θ∂X+V∂θ∂Y−∂2θ∂X2−∂2θ∂Y2)+∫HS1α¯w3(θ−θHS1)dΓ+∫HS2α¯w3(θ−θHS2)dΓ=0,

where *w*_1_, *w*_2_, *w*_3_ are to be replaced by the MLS shape functions Φ_*I*_  (*I* = 1,2,…, *n*) for obtaining stiffness matrix expressions. $\bar{\alpha }$ is the penalty parameter imposed to enforce the essential boundary conditions. The values of *U*, *V*, *θ* along the boundaries are prescribed in Figure 1 and (7)–(9).

Using the EFGM model given by (25), into (26), the system of equations can be defined as follows:

(27) $$\begin{array}{c} \left[\begin{array}{ccc} K_{11} & K_{12} & K_{13}\\ K_{21} & K_{22} & K_{23}\\ K_{31} & K_{32} & K_{33} \end{array}\right]\left[\begin{array}{c} U\\ V\\ \theta \end{array}\right]=\left[\begin{array}{c} H_{1}\\ H_{2}\\ H_{3} \end{array}\right], \end{array}$$

where each of these [*K*_*rs*_], [*H*_*s*_], (*r*, *s* = 1,2, 3) are given as follows:

(28) (K11)IJ=∫Ω(ΦIΦJ∂U¯∂X+γ∂ΦI∂X∂ΦJ∂X+Pr∂ΦI∂X∂ΦJ∂X+Pr∂ΦI∂Y∂ΦJ∂Y)dX dY+∫Γ1α¯ΦIΦJdΓ+∫Γ2α¯ΦIΦJdΓ+∫Γ3α¯ΦIΦJdΓ+∫Γ4α¯ΦIΦJdΓ,(K12)IJ=∫Ω(ΦIΦJ∂U¯∂Y+γ∂ΦI∂X∂ΦJ∂Y)dX dY,(K13)IJ=0,(K21)IJ=∫Ω(ΦIΦJ∂V¯∂X+γ∂ΦI∂Y∂ΦJ∂X)dX dY,(K22)IJ=∫Ω(ΦIΦJ∂V¯∂Y+γ∂ΦI∂Y∂ΦJ∂Y+Pr∂ΦI∂X∂ΦJ∂X+Pr∂ΦI∂Y∂ΦJ∂Y)dX dY+∫Γ1α¯ΦIΦJdΓ+∫Γ2α¯ΦIΦJdΓ+∫Γ3α¯ΦIΦJdΓ+∫Γ4α¯ΦIΦJdΓ,(K23)IJ=∫Ω−Ra Pr ΦIΦJdX dY,(K31)IJ=∫Ω(ΦIΦJ∂θ¯∂X)dX dY,(K32)IJ=∫Ω(ΦIΦJ∂θ¯∂Y)dX dY,(K33)IJ=∫Ω(∂ΦI∂X∂ΦJ∂X+∂ΦI∂Y∂ΦJ∂Y)dX dY+∫HS1α¯ΦIΦJdΓ+∫HS2α¯ΦIΦJdΓ,

(29) (H1)I=∫Γ1α¯UΓ1ΦIdΓ+∫Γ2α¯UΓ2ΦIdΓ+∫Γ3α¯UΓ3ΦIdΓ+∫Γ4α¯UΓ4ΦIdΓ,(H2)I=∫Γ1α¯VΓ1ΦIdΓ+∫Γ2α¯VΓ2ΦIdΓ+∫Γ3α¯VΓ3ΦIdΓ+∫Γ4α¯VΓ4ΦIdΓ,(H3)I=∫HS1α¯θHS1ΦIdΓ+∫HS2α¯θHS2ΦIdΓ,U¯=∑I=1n∗ΦIU¯I,  V¯=∑I=1n∗ΦIV¯I,  θ¯=∑I=1n∗ΦIθ¯I,

where *I*, *J* = (1,2,…, N*) and N* denotes the total number of nodes in the whole problem domain.

### 3.6. Background Integration

The whole domain *Ω* is discretized with 41 × 41, that is, 1681 nodes in such a manner that nodes are denser near the cold wavy surface as compared to the hot flat surface. For numerical integration purpose, a background mesh of size 40 × 40 is utilized and four-point Gauss quadrature formula has been used to evaluate the viscous terms in [*K*_11_], [*K*_12_], [*K*_21_], [*K*_22_], whereas for the evaluation of penalty terms in [*K*_11_], [*K*_12_], [*K*_21_], [*K*_22_] the reduced Gauss integration scheme that is one-point Gauss quadrature formula has been used. Large values of penalty parameter *γ*, can satisfy the constraint condition or continuity condition absolutely, but due to high penalty parameter *γ*, the contribution from viscous terms in [*K*_11_], [*K*_12_], [*K*_21_], [*K*_22_] (given in (28)) becomes negligible as compare to the penalty terms. Hence, it implies that, for infinitely large values of penalty parameter *γ*, the governing equations are left only with constraint condition or continuity condition and the contribution from momentum and energy conservation equations is completely lost. In order to avoid such a situation, reduced Gauss integration scheme [24] has been applied for the evaluation of penalty terms in [*K*_11_], [*K*_12_], [*K*_21_], [*K*_22_].

At each node, three functions *U*, *V*, *θ* are to be evaluated; hence after assembly, we obtain a nonlinear system of equations of order 5043 × 5043, as given in (27). Owing to the nonlinearity of the system, an iterative scheme has been used to solve it with an initial guess. The system of equations is linearized by incorporating known functions $\bar{U}$ , $\bar{V}$ , $\bar{\theta }$ as given in (29), which is solved using Gauss elimination method. This gives a new set of values of unknowns *U*, *V*, *θ* and the process continues till the required accuracy (0.0005) is achieved.

### 3.7. Validation of the Results

**Figure 3:**
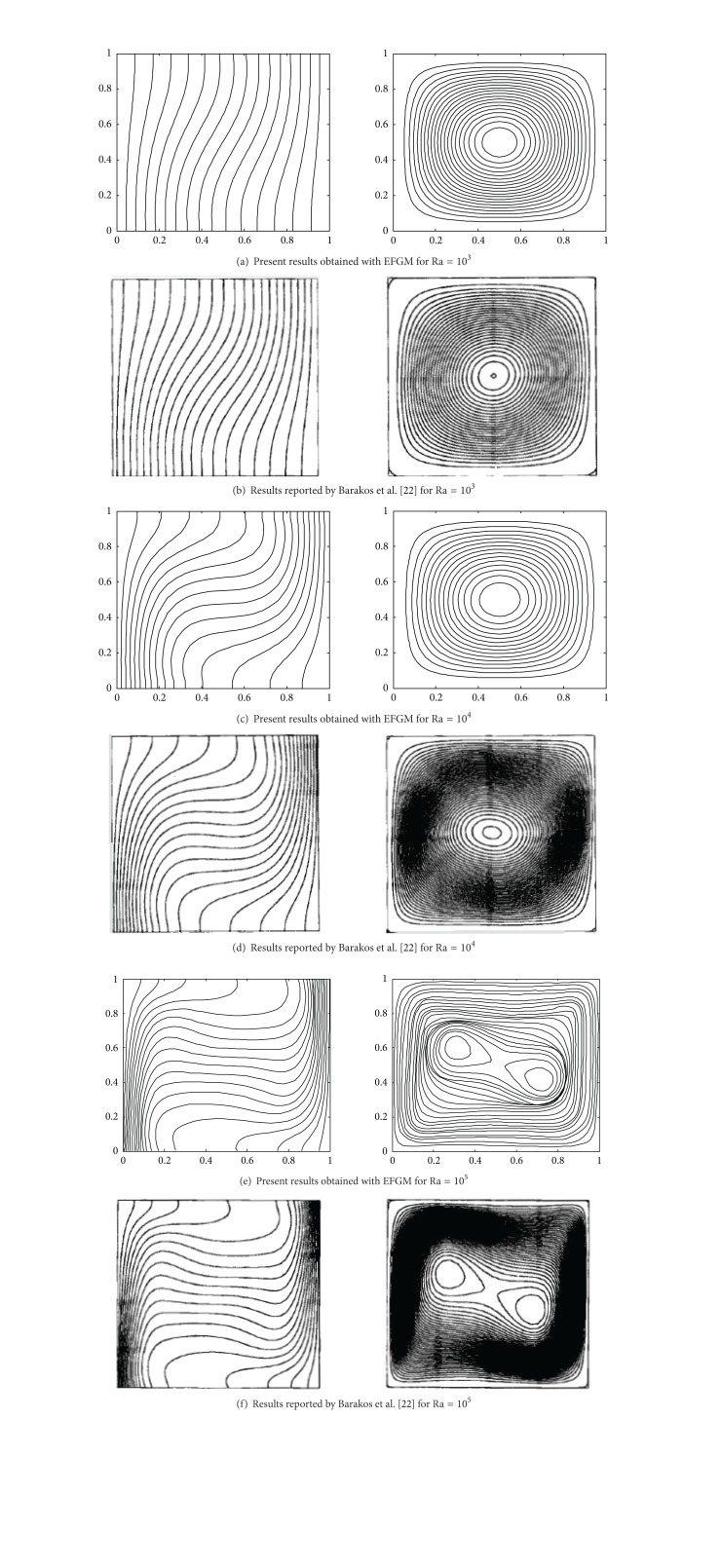
Comparison of streamlines and isotherms profile with benchmark results reported by Barakos et al. [22].

**Figure 4:**
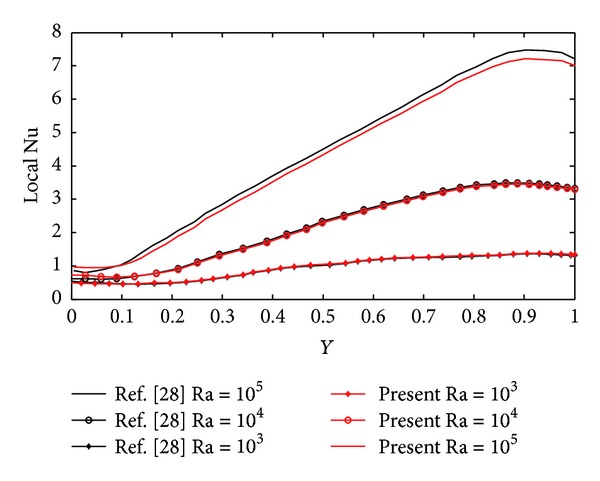
Comparison of spatial structure and variation of nusselt number along cold surface.

We have validated our results for a canonical problem available in the literature reported by De Vahl Davis [2], Barakos et al. [22], Fusegi et al. [23], and Wan et al. [28] in Figures 3 and 4. The spatial structure of local nusselt number and mean nusselt number obtained with present EFGM technique is compared with benchmark results in Figure 4 and Table 1, respectively, and a good agreement has been observed between them.

## 4. Results and Discussions

In the present work, numerical calculations are carried out over a wide range of parameters, Rayleigh number, Ra  (10^4^ ≤ Ra ≤ 5 × 10^5^), Prandtl number, Pr  (0.07 ≤ Pr ≤ 7), and dimensionless length of the heaters in the *x*- and *y*-direction (0.2 ≤ *hx* ≤ 0.8,  0.2 ≤ *hy* ≤ 0.8) to investigate their impact on thermal and flow fields. The flow and temperature fields are graphically presented in terms of stream lines and isotherm contours, respectively. Heat transfer characteristics are examined in terms of mean and local nusselt numbers at the cold wavy surface and corner heaters.

### 4.1. Effect of Rayleigh Number

**Figure 5:**
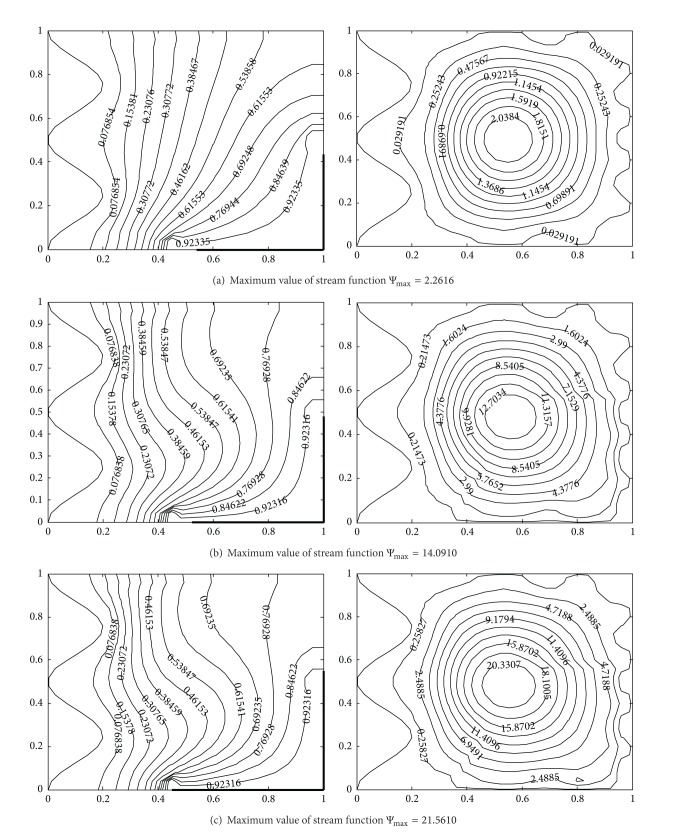
Temperature contours (on the left) and streamlines (on the right) with different Rayleigh number at Pr = 0.7, *A* = 0.2, *n* = 2, *hx* = 0.55, *hy* = 0.55, (a) Ra = 10^4^, (b) Ra = 10^5^, and (c) Ra = 5 × 10^5^.

The impact of Rayleigh number on flow and temperature field is represented in terms of streamlines and isotherms, respectively, in Figure 5. From the contours of the isotherms, it is observed that the dominant heat transfer mechanism changes from conduction to convection with increase in Ra, due to increased buoyancy. For low Ra-values the heat is transferred by conduction between hot wall and the cold wavy wall because these almost vertical isotherms lines are more concentrated in the vicinity of hot corner. For high Ra-values, the heat transfer mechanism changes from conduction to convection and the isotherm lines depart from the hot corner towards the cold wavy surface. For higher values of Rayleigh number, convection strength is increased. Comparing the results shown in Figure 3 for higher Ra-values, with the results shown in Figure 5, it could be noticed that isotherms in Figure 3 at the centre of the cavity are becoming almost horizontal showing dominant convection heat transfer mechanism which is not so in isotherm contours of Figure 5, because heating is being done on a part of the vertical and horizontal side of cavity. Therefore, for controlled heat transfer partial heaters could be used in many physical applications.

From the stream function plots in Figure 5 for 10^4^–5 × 10^5^, it can be seen that fluid is unicellular and rotates in counterclockwise direction. For low Ra-values, a single circulating eddy appears as the dominant characteristic of the flow. The magnitude of the stream functions increases significantly with increase in Ra from 10^4^–5 × 10^5^. Hence, higher flow strength is observed for higher values of Ra since Ra increases the buoyancy effect. Due to asymmetric heating, higher values of the velocities are observed in the region of corner heaters as compared to the velocities obtained in the upper half (insulated part) of the enclosure and it gives more intended streamline towards the heated corner.

### 4.2. Effect of Prandtl Number

**Figure 6:**
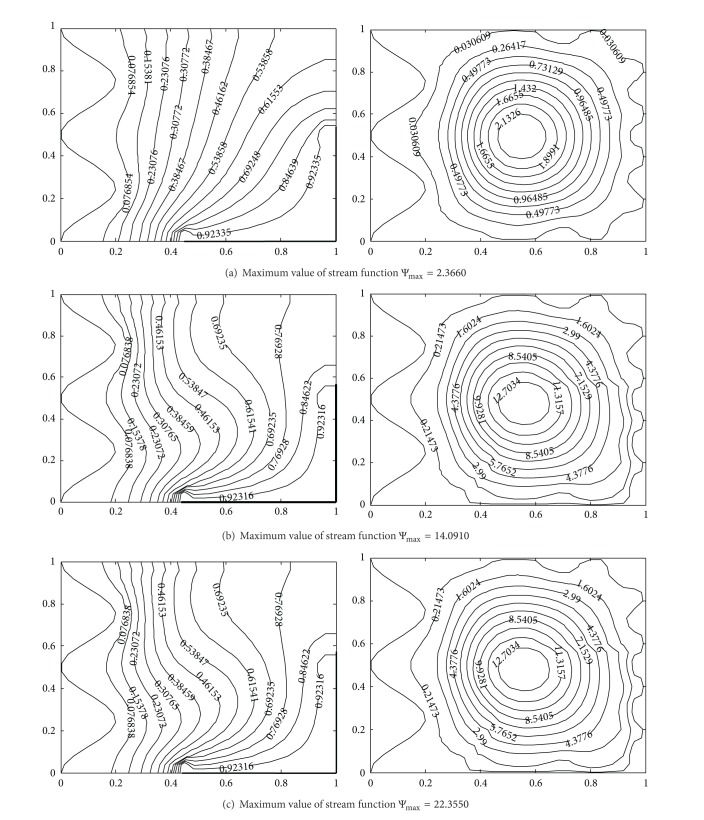
Temperature contours (on the left) and streamlines (on the right) with different Prandtl number at Ra = 10^5^, *A* = 0.2, *n* = 2, *hx* = 0.55, *hy* = 0.55, (a) Pr = 0.07, (b) Pr = 0.7, and (c) Pr = 7.0.

Figure 6 depicts the impact of Prandtl number on isotherms and streamlines (for Pr = 0.07–7). For the lowest Prandtl number (Pr = 0.07), the stream function values are very low and isotherm lines are more concentrated towards the hot corner. However, for higher Prandtl number, isotherms are denser near the cold wavy side wall and higher values of stream functions are observed. In comparison with, considering the contour plots of Pr = 7.0 with Pr = 0.7, we observe that they look qualitatively similar which shows that the nature of streamlines and isotherms does not change considerably with higher Prandtl number. However, the maximum stream function values are obtained with Pr = 7.0.

### 4.3. Effect of Corner Heating

**Figure 7:**
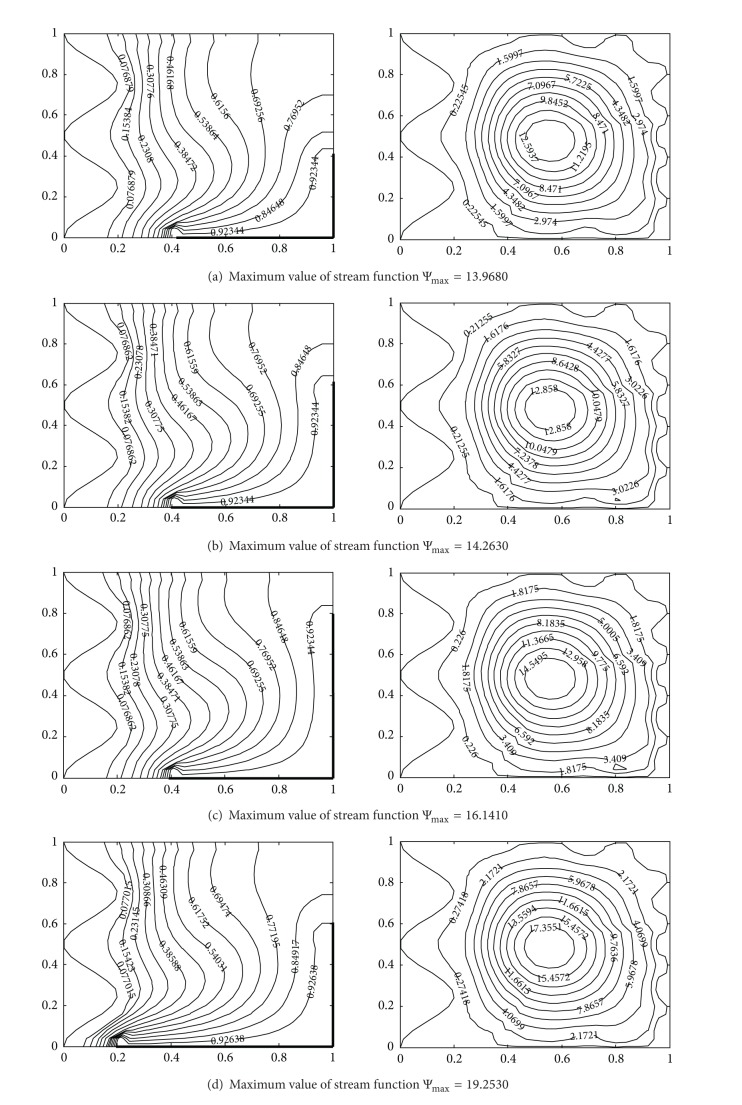
Temperature contours (on the left) and streamlines (on the right) with different heater length at Ra = 10^5^, Pr = 0.7, *A* = 0.2, *n* = 2, (a) *hx* = 0.6, *hy* = 0.4, (b) *hx* = 0.6, *hy* = 0.6, (c) *hx* = 0.6, *hy* = 0.8, and (d) *hx* = 0.8, *hy* = 0.6.

The impact of heater length on flow and temperature field is illustrated in Figure 7 for Ra = 10^5^, Pr = 0.7. In Figures 7(a)–7(c), the horizontal length of the heater, that is, *hx*, is kept fixed at 0.6 and, results are obtained with different values of vertical heater length, that is, *hy* = 0.4–0.8. From these contours, higher temperature gradient near the heater is observed with increase in heater length in vertical direction due to stronger convection and therefore heat transfer rate increases with increase in heater length. In Figure 7(d), higher heater length is chosen in horizontal direction (*hx* = 0.8 and *hy* = 0.6). It shows steep temperature gradient near the heater due to stronger convection and shape of the isotherms shows plume-like distribution. Comparing Figure 7(c) (*hx* = 0.6 and *hy* = 0.8) with (d) (*hx* = 0.8 and *hy* = 0.6), higher values of stream function are obtained for *hx* = 0.8 and *hy* = 0.6. It indicates that given the same total heater length, the flow strength is increased with an increase of the length in horizontal direction. At the centre of the enclosure, the highest values of the stream functions are obtained.

### 4.4. Effect of Wavy Surface Amplitude and Number of Undulations

**Figure 8:**
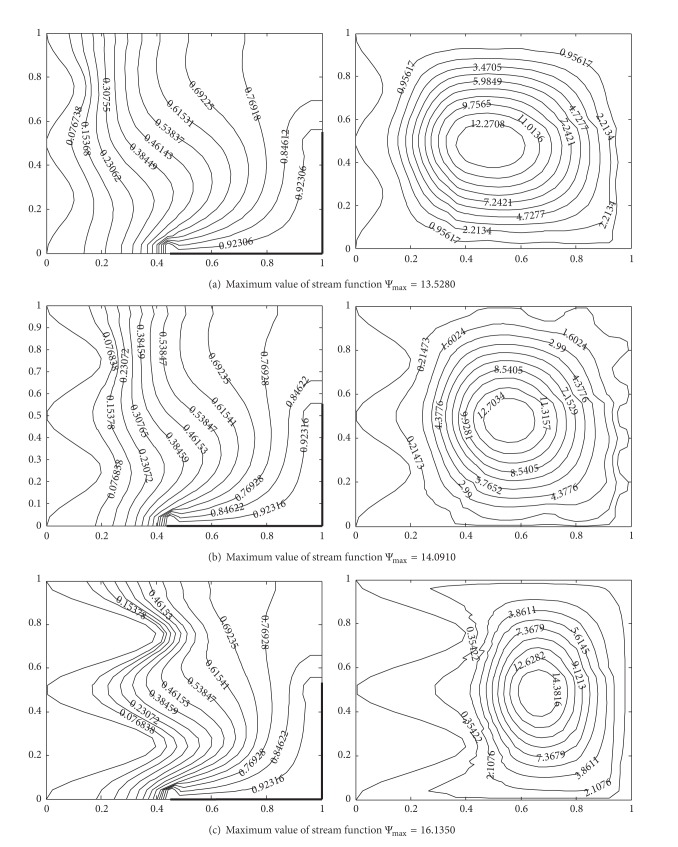
Temperature contours (on the left) and streamlines (on the right) with different wavy surface amplitudes at Ra = 10^5^, Pr = 0.7, *n* = 2, *hx* = 0.55, *hy* = 0.55, (a) *A* = 0.1, (b) *A* = 0.2, and (c) *A* = 0.4.

**Figure 9:**
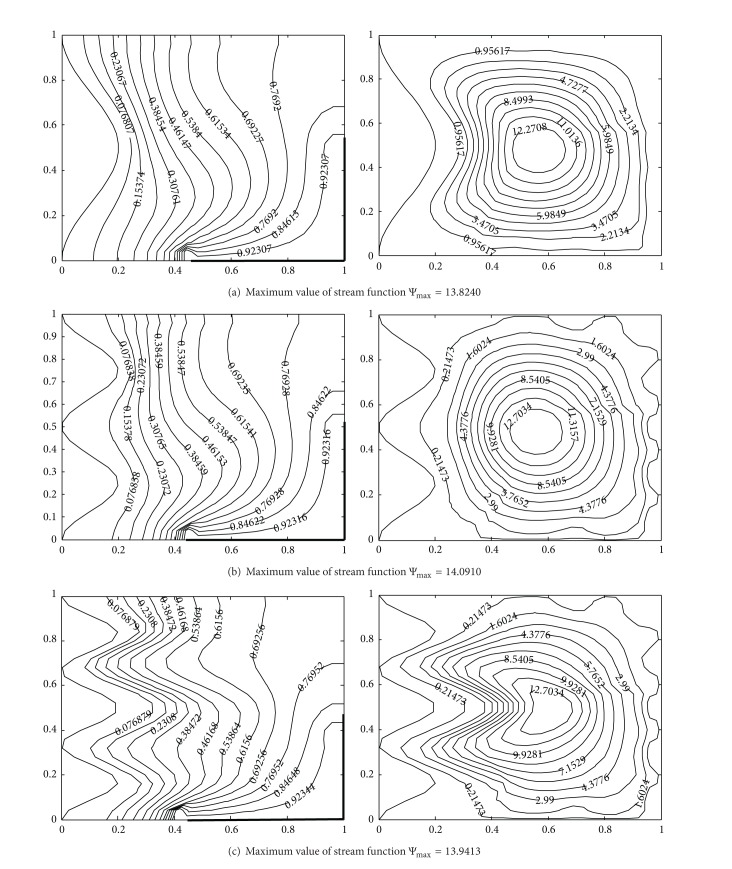
Temperature contours (on the left) and streamlines (on the right) with different numbers of undulations of wavy surface at Ra = 10^5^, Pr = 0.7, *A* = 0.2, *hx* = 0.55, *hy* = 0.55, (a) *n* = 1, (b) *n* = 2, and (c) *n* = 3.

In Figure 8 contour plots of stream lines and isotherms are shown for Ra = 10^5^, Pr = 0.7, with different wavy amplitudes (*A* = 0.1–0.4). In Figure 9, contours of stream lines and isotherms are drawn for Ra = 10^5^, Pr = 0.7 and wavy surface amplitude *A* = 0.2 with different numbers of undulation (*n* = 1, 2,3). From Figure 8, it is observed that with increase in wavy surface amplitude, the inward protrusion of the isotherm lines increases. This narrows down the straight vertical passage for the fluid to move under the influence of buoyancy force and the isotherms show the steep temperature gradient near the wavy surface. Due to which higher heat transfer rate along the cold wavy surface is observed with increased wavy surface amplitude. Therefore, rough surfaces can be used for higher heat transfer rate while designing physical devices. It appears from Figures 9(a)–9(c) that varying the number of undulations does not change the global flow and isotherms pattern except in the vicinity of the vertical wavy wall. In the vicinity of the vertical wavy wall, the isotherm lines and stream lines adopt the profile of wavy wall. Increment in the number of undulations along the wavy surface has a negative impact on the heat transfer rate. Heat transfer rate is decreased with the increase of number of undulations but not significantly.

### 4.5. Heat Transfer Characteristics

**Figure 10:**
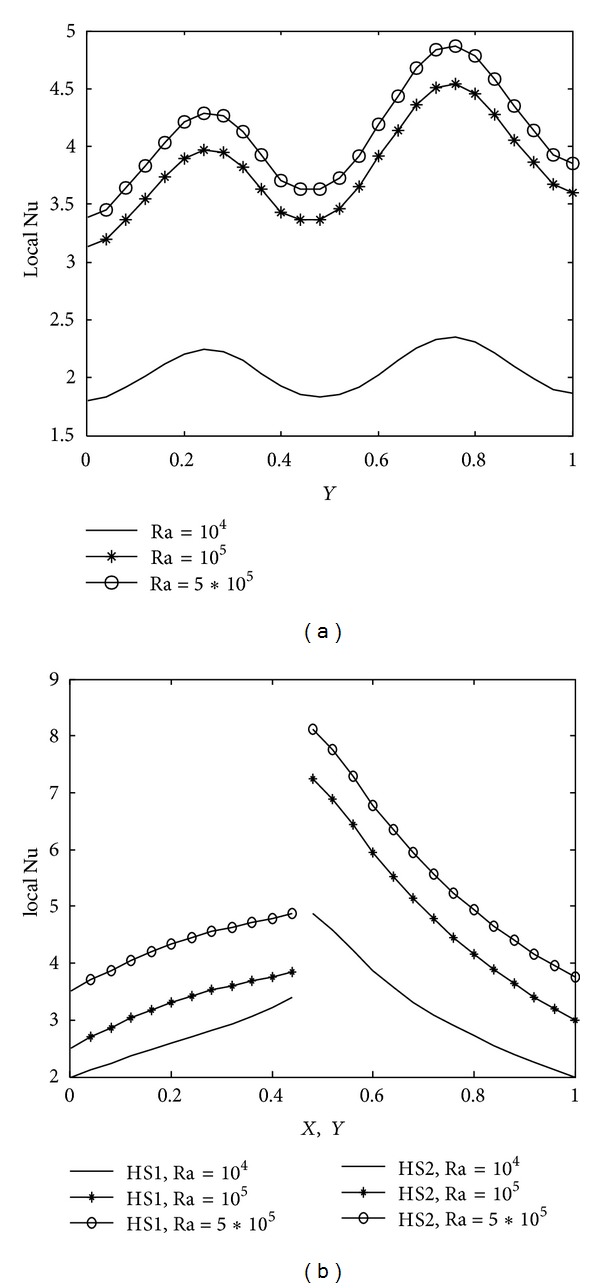
Variation of local nusselt number with Ra at Pr = 0.7, *A* = 0.2, *n* = 2, *hx* = 0.55, and *hy* = 0.55 (a) along cold wavy surface and (b) along the heaters.

**Figure 11:**
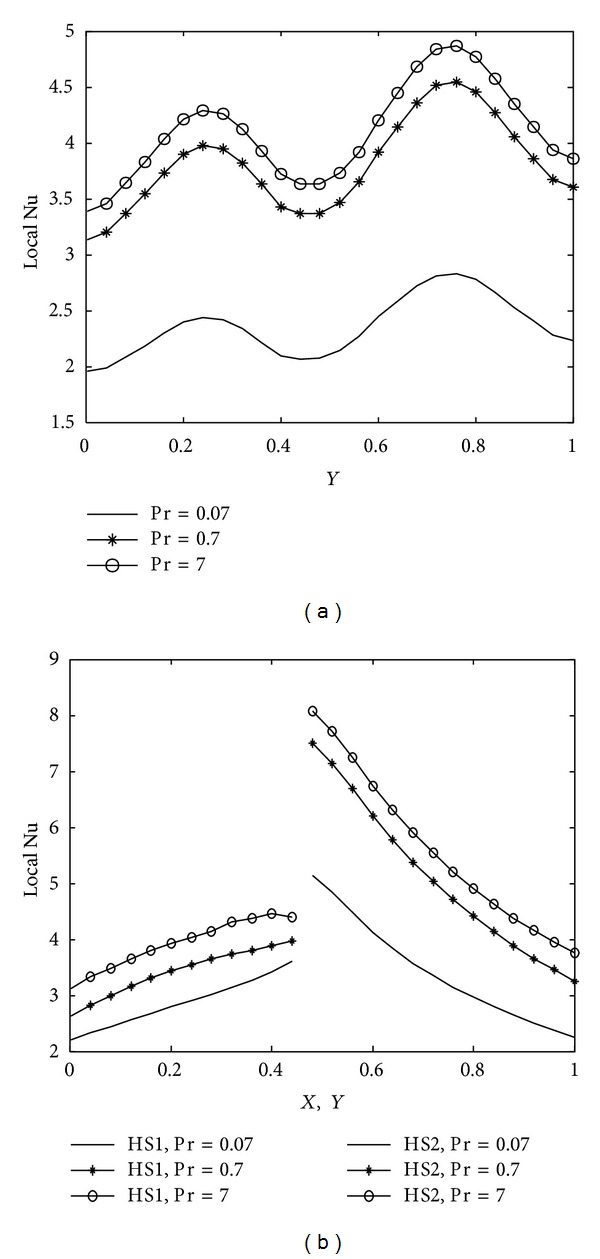
Variation of local nusselt number with Pr at Ra = 10^5^, *A* = 0.2, *n* = 2, *hx* = 0.55, and *hy* = 0.55 (a) along cold wavy surface and (b) along the heaters.

**Figure 12:**
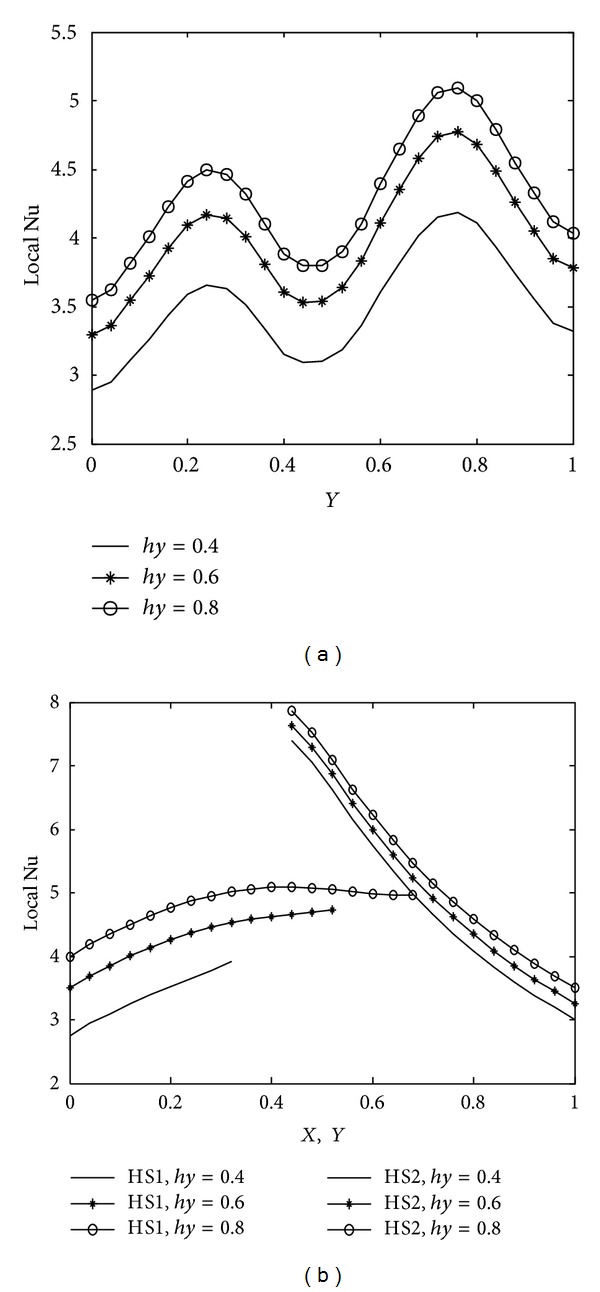
Variation of local nusselt number with heater length in vertical direction *hy* at Ra = 10^5^, Pr = 0.7, *A* = 0.2, *n* = 2, and *hx* = 0.6 (a) along cold wavy surface and (b) along the heaters.

**Figure 13:**
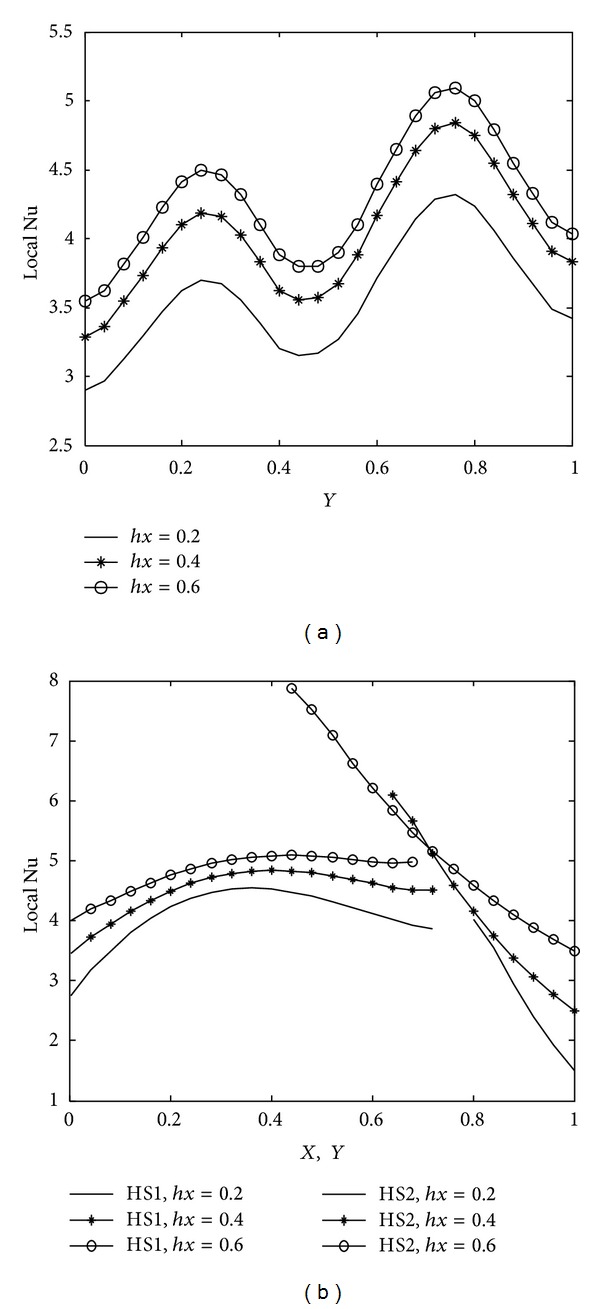
Variation of local nusselt number with heater length in horizontal direction at Ra = 10^5^, Pr = 0.7, *A* = 0.2, *n* = 2, and *hy* = 0.8 (a) along cold wavy surface and (b) along the heaters.

**Figure 14:**
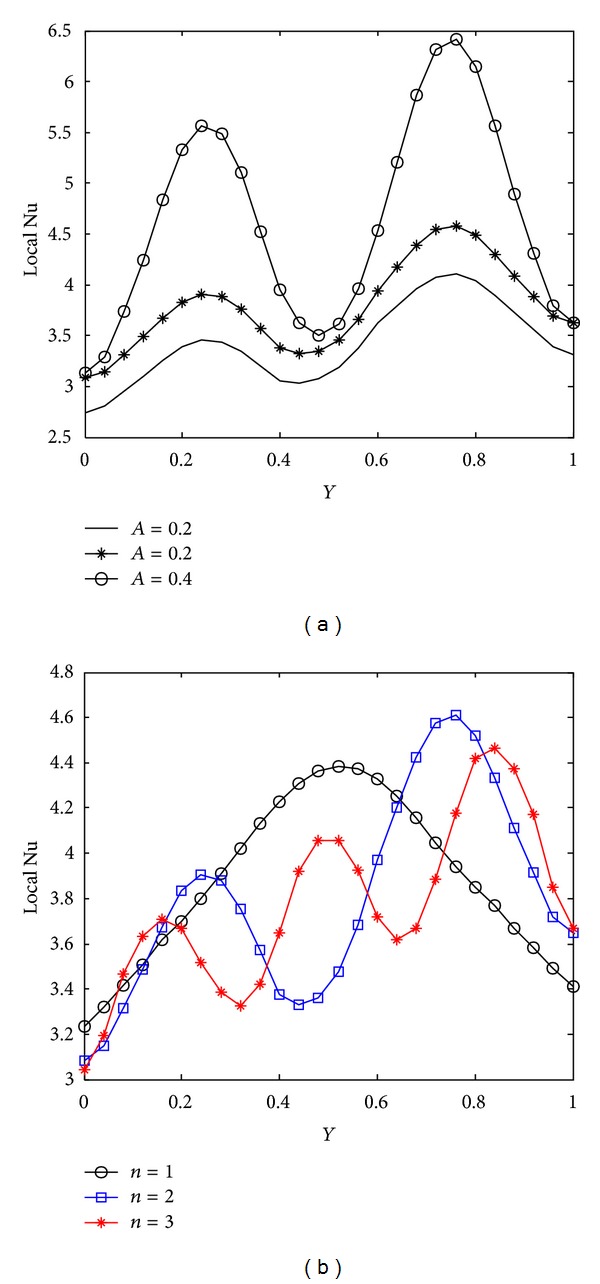
Variation of local nusselt number with Ra = 10^5^, Pr = 0.7, *hx* = 0.55, and *hy* = 0.55 at cold wavy surface (a) with wavy surface amplitude (*A*) and (b) with number of undulations (*n*).

The implications of various parameters, Rayleigh number, Prandtl number, heater length, wavy surface amplitude and number of undulations on energy transport process, are reported in terms of mean and local nusselt number along the cold wavy wall and corner heaters. The average nusselt number obtained along the cold wavy surface is given in Table 2 for all the parameters and spatial variation of the nusselt number for different parameters is depicted in Figures 10–14.

Due to wavy geometry, local nusselt number profiles along the cold wavy surface are also wavy in nature.  Figure 10 indicates that heat transfer rate increases rapidly for higher Rayleigh number due to strong convection. The smallest value of the mean nusselt number is obtained with Ra = 10^4^.  Figure 11 shows the variation of heat transfer rate with prandtl number. We observe from Figure 11 that prandtl number is more significant on heat transfer rate for Pr < 1 and that the difference between heat transfer rate obtained with Pr = 0.07 and Pr = 0.7 is much bigger as compared to the heat transfer rate calculated with Pr = 0.7 and Pr = 7.0. Heater length both in vertical and horizontal directions has considerable impact on heat transfer rate, as shown in Figures 12 and 13. At the intersection point of the heaters, the highest value of nusselt number is obtained. Higher heat transfer rate is observed with increased heater length in vertical or horizontal direction.  Figure 14 depicts the impact of wavy surface amplitude and number of undulations on heat transfer rate. Both average nusselt number along the cold way wall and local nusselt number are higher for higher wavy amplitude. It shows that heat transfer rate is higher for wavy surface as compared to the smooth surface while the impact of the number of undulations is not so significant on average nusselt number. A slight decrease in the heat transfer rate is observed with more undulations along the wavy surface.

## 5. Conclusions

The problem of natural convection heat transfer and fluid flow within a wavy enclosure with corner heating effect has been studied. The meshfree element free Galerkin model is demonstrated as an alternative approach to eliminate the well known drawbacks (meshing and remeshing) of grid based methods such as FDM and FEM. Obtained results are verified with available benchmark results and a good agreement has been observed. It is observed that Rayleigh number has significant impact on heat transfer rate and heat transfer rate is an increasing function of Rayleigh number. Another significant parameter which influences the heat transfer is the heater's length in vertical and horizontal directions. However, given the same total heater length, the flow strength is increased with an increase of the length in horizontal direction. The impact of increasing prandtl number is remarkable on flow, temperature, and heat transfer for Pr < 1. Heat transfer rate increases with increase of prandtl number. The shape of the heat transfer surface also plays an important role. Heat transfer rate is higher for wavy surface as compared to the smooth surface.

## Figures and Tables

**Table 1:** Comparison of mean Nusselt obtained by present technique with benchmark solutions of De Vahl Davis [2], Barakos et al. [22], and Fusegi et al. [23] for natural convection within a square cavity.

Ra	10^3^	10^4^	10^5^
Nu [2]	1.118	2.243	4.519
Nu [22]	1.114	2.245	4.510
Nu [23]	1.105	2.302	4.646
Nu (present) with 17 × 17 nodes	1.016	2.239	4.499
Nu (present) with 26 × 26 nodes	1.119	2.248	4.528
Nu (present) with 41 × 41 nodes	1.119	2.250	4.535

**Table 2:** Mean Nusselt number computed along the cold wavy surface for different parameters.

Rayleigh number (Ra)	Prandtl number (Pr)	Heater length in the *x*-direction (*h*_*x*_)	Heater length in the *y*-direction (*h*_*y*_)	Wavy surface amplitude (*A*)	Number of undulations (*n*)	Mean Nusselt number (Nu_avg_)
10^4^	0.7	0.55	0.55	0.2	2	2.0547
10^5^	0.7	0.55	0.55	0.2	2	3.8043
5 × 10^5^	0.7	0.55	0.55	0.2	2	4.0922
10^5^	0.07	0.55	0.55	0.2	2	2.3555
10^5^	0.7	0.55	0.55	0.2	2	3.8043
10^5^	7.0	0.55	0.55	0.2	2	4.0945
10^5^	0.7	0.6	0.4	0.2	2	3.5028
10^5^	0.7	0.6	0.6	0.2	2	3.9955
10^5^	0.7	0.6	0.8	0.2	2	4.2844
10^5^	0.7	0.2	0.8	0.2	2	3.5798
10^5^	0.7	0.4	0.8	0.2	2	4.0293
10^5^	0.7	0.6	0.8	0.2	2	4.2844
10^5^	0.7	0.55	0.55	0.1	2	3.4196
10^5^	0.7	0.55	0.55	0.2	2	3.8043
10^5^	0.7	0.55	0.55	0.4	2	4.5358
10^5^	0.7	0.55	0.55	0.2	1	3.8773
10^5^	0.7	0.55	0.55	0.2	2	3.8043
10^5^	0.7	0.55	0.55	0.2	3	3.7686

## Nomenclature

*g*: gravitational acceleration m/s^2^
*N*: normal direction on the plate
Ra: Rayleigh number
Pr: Prandtl Number
Nu: mean nusselt number
*a*: wavy surface amplitude
*n*: number of undulations
*T*: temperature, K
*hx*: dimensionless heater length in the *x*-direction
*hy*: dimensionless heater length in the *y*-direction
*u*, *v*: velocities in the *x*- and *y*-direction, m/s
*U*, *V*: nondimensional velocities in the *x*- and *y*-direction
*X*, *Y*: nondimensional coordinates
*L*: side of the cavity, m
HS1: heated surface 1 (horizontal)
HS2: heated surface 2 (vertical)
CS: cold surface.

### Greek Letters

*υ*: kinematic viscosity, m^2^ s^−1^
*α*: thermal diffusivity, m^2^ s^−1^
*θ*: nondimensional temperature
*β*: thermal expansion coefficient, K^−1^
*ψ*: nondimensional stream function.

### Subscripts

*c*: cold
*h*: hot.
